# Quality of chest compressions during pediatric resuscitation with 15:2 and 30:2 compressions-to-ventilation ratio in a simulated scenario

**DOI:** 10.1038/s41598-020-63921-9

**Published:** 2020-04-22

**Authors:** Gema Manrique, Araceli González, Maitane Iguiñiz, Ana Grau, Blanca Toledo, Miriam García, Jesús López-Herce

**Affiliations:** 1 Pediatric Intensive Care Department, Gregorio Marañon University Hospital, Madrid, Spain; 2 Public Health, Maternal and Child Department, School of Medicine, Complutense University of Madrid, Madrid, Spain; 3 Health Research Institute of the Gregorio Marañón Hospital, Madrid, Spain; 4 Research Network on Maternal and Child Health and Development (RedSAMID), Madrid, Spain; 5 Pediatric Department, Gregorio Marañon University Hospital, Madrid, Spain

**Keywords:** Diseases, Medical research

## Abstract

The main objetive was to compare 30:2 and 15:2 compression-to-ventilation ratio in two simulated pediatric cardiopulmonary resuscitation (CPR) models with single rescuer. The secondary aim was to analyze the errors or omissions made during resuscitation. A prospective randomized parallel controlled study comparing 15:2 and 30:2 ratio in two manikins (child and infant) was developed. The CPR was performed by volunteers who completed an basic CPR course. Each subject did 4 CPR sessions of 3 minutes each one. Depth and rate of chest compressions (CC) during resuscitation were measured using a Zoll Z series defibrillator. Visual assessment of resuscitation was performed by an external researcher. A total of 26 volunteers performed 104 CPR sessions. Between 54–62% and 44–53% of CC were performed with an optimal rate and depth, respectively, with no significant differences. No differences were found in depth or rate of CC between 15:2 and 30:2 compression-to-ventilation ratio with both manikins. In the assessment of compliance with the ERC CPR algorithm, 69.2–80.8% of the subjects made some errors or omissions during resuscitation, the most frequent was not asking for help and not giving rescue breaths. The conclusions were that a high percentage of CC were not performed with optimal depth and rate. Errors or omissions were frequently made by rescuers during resuscitation.

## Introduction

Pediatric cardiac arrest (CA) is an important health problem since it has high mortality (52–80%) and a large proportion of survivors suffer from permanent and severe neurologic disability (poor outcome in 20–50%)^1–4^. Previous studies have shown that optimizing maneuvers of cardiopulmonary resuscitation (CPR) in children could improve survival and prognosis^4,5^. Nevertheless, many studies documented that CPR is often not optimally performed^6,7^.

The 2015 European Resuscitation Council (ERC) guidelines recommend a synchronized 15:2 compression-to-ventilation ratio during basic pediatric CPR^1,8,9^. However, there is not a strong evidence to indicate the best compression-to-ventilation ratio in pediatric CPR^10,11^. Firstly, maintaining an adequate ventilation is crucial during CPR in children because pediatric CA is primarily caused by respiratory failure^2^. The 15:2 ratio delivers more ventilations whereas the 30:2 ratio delivers more chest compressions (CC). In the other hand, different recommendations for the compression-to-ventilation ratio between children (15:2 ratio) and adults (30:2 ratio) could increase the errors or omissions and impair learning. In adult simulation models^12–14^, there are different studies that compare the quality of CC in both compression-to-ventilation ratio. However, only one study conducted in pediatric models, but it was developed by volunteers with pediatric advanced life support (PALS) accreditation^15^. In our knowledge, there are no previous studies comparing the quality of CC between 15:2 and 30:2 compression-to-ventilation ratio during pediatric CPR conducted by volunteers training in basic life support.

### Hypothesis and objectives

The hypothesis of the study is that the quality of chest compressions is the same with 15:2 as 30:2 ventilation-to-compression ratio in a pediatric simulation model. For this purpose, the main objective was to compare the quality of chest compressions and performance of single rescuer CPR comparing two compressions-to-ventilation ratios 15:2 and 30:2 in a simulated scenario with two manikins (infant and child model). The secondary aim was to describe the visual assessment of the quality of resuscitation in both scenarios.

## Methods

### Design

We designed a prospective randomized parallel controlled study of simulated pediatric CPR to compare the depth and rate of CC using a 15:2 vs 30:2 compression-to-ventilation ratio in manikins. The CPR was performed by medical students and pediatric residents and two manikins were used: infant and child model. The study was approved by the Gregorio Marañón Ethics Committee of Madrid, Spain.

### Participants

Twenty-six volunteer senior medical students who recently completed a basic pediatric CPR course. All participants provided their informed consent.

### Equipment and materials

Two CPR training manikins were used: Resuscijunior and Resuscibaby (Laerdal, Wappingers Falls, NY, USA). CPR electrodes were applied onto sternum and connected to a Zoll R Series Monitor/Defibrillator (ZOLL Medical Corporation, Chelmsford, MA, USA) to record CPR-quality parameters.

### Intervention

Before beginning CPR, participants received a practical demonstration of pediatric and infant basic life support algorithm in both manikins by one of the senior researchers. All participants could practice chest compressions and ventilation in both manikins prior to CPR session.

Each rescuer performed four CPR sessions, of three minutes duration each of them, with a resting period of at least 5 minutes between each period. Two of the CPR sessions were performed on the infant manikin and two on the child manikin. Two CPR sessions with different compression-to-ventilation ratio (30:2 and 15:2) were developed on each manikin. The order of beginning each session were randomized.

Child CPR was delivered while kneeling beside the manikin on the floor. On the infant manikin, an encircling technique was used for chest compressions with the manikin lying on a table^1^.

For child manikin, compression depth between 3.8 and 5.1 centimeters (cm) and a rate between 100 and 120 min^−1^ were considered optimal. For the infant manikin optimal compression depth was between 3 cm and 4 cm.

The rescuers did not receive feedback of depth and rate of CC from the monitor during CPR sessions.

### Measurements

Three researchers trained in pediatric cardiopulmonary resuscitation participated in CPR sessions. One researcher coordinated CPR sessions, other researcher made sure that the defibrillator measured properly and the third researched visually assessed whether the maneuvers were properly performed.

Data measured and stored in Zoll defibrillator were analyzed with RescueNet Code Review program. The following variables over one-minute intervals were analyzed: depth and rate of CC, release velocity (mm/s), time without CC and the percentage of optimal compressions (in depth and rate).

Video recordings of the CPR sessions were performed to check the researcher’s visual assessment of the overall quality of resuscitation. One of the researchers (always the same one) checked whether the basic life support algorithm and maneuvers were satisfactorily followed by each rescuer. The opening of the airway was considered inadequate if any of these events occurred: in the child manikin the rescuer did not perform a good neck extension or did not pinch the nose, and, in the infant manikin, the rescuer did not have a good placement of the hands in the front-chin maneuver. Ventilation maneuver was inadequate when the observer researcher considered it to be too fast, too slow, or excessive or shallow chest rise. Other items assessed were whether the rescuer requested help, if they checked breathing or pulse and the accomplishment of the order of the basic life support algorithm.

At the end of all sessions, participants were asked which compression-ventilation ratio (15:2 or 30:2) they preferred in each manikin.

### Statistical analysis

The SPSS statistical package, version 20.0 (SPSS Inc, Chicago, USA) was used for statistical analysis. Normal distribution of variables was tested with the Kolmogorov-Smirnov test. Continuous variables are expressed as mean and standard deviations or medians with interquartile ranges and categorical variables as percentages. Paired Student t-test was used to compare continuous variables. McNemar’s test was used to compare categorical variables with paired data. P values less than 0.05 were considered significant.

### Statement

All methods were carried out in accordance with relevant guidelines and regulations.

## Results

Twenty-six volunteers, 53.8% men, performed 104 resuscitation sessions (four per rescuer). All of them had previously completed a pediatric CPR course, 7.8 ± 1.0 months before the study.

### Parameters of CPR quality

Table 1 showed CC rate in the infant and child manikin (including variables of data collection sheet and those analyzed by the program RescueNet Code Review). Only 59.6% and 54.9% of the CC, respectively, were performed with a guideline recommended rate. There were no differences in the rate of CC between the ratio 15:2 and 30:2.

**Table 1:** Rate of chest compressions.

Parameters		Children manikin			Infant manikin		
		15:2 ratio	30:2 ratio	p	15:2 ratio	30:2 Ratio	p
		(n = 26)	(n = 26)		(n = 26)	(n = 26)	
Mean rate (min-^1^) Mean (SD)	Minute 1	109.0 (9.2)	109.0 (11.8)	0.98	106.3 (10.7)	107.8 (11.4)	0.54
	Minute 2	109.3 (11.1)	108.2 (12.3)	0.63	106.2 (10.4)	107.3 (11.9)	0.64
	Minute 3	108.9 (10.4)	108.2 (11.8)	0.74	105.4 (12.1)	106.6 (13.0)	0.64
	Overall	109.1 (11.5)	108.5 (12.7)	0.78	108.3 (13.7)	107.2 (12.4)	0.68
Percentage of chest compressions Mean (SD)	Lower rate than optimal	14.3 (22.6)	20.3 (30.6)	0.35	18.5 (21.0)	23.8 (33.2)	0.39
	Optimal rate	55.8 (35.1)	54.1 (39.1)	0.83	62.1 (31.5)	57.0 (31.1)	0.55
	Higher rate than optimal	29.4 (34.5)	28.8 (40.1)	0.94	19.4 (31.5)	19.2 (25.8)	0.98
Number of chest compressions Mean (SD)	Minute 1	54.4 (10.4)	67.5 (12.4)	**<0.01**	56.1 (9.5)	67.3 (12.2)	**<0.01**
	Minute 2	64.0 (11.9)	81.0 (10.7)	**<0.01**	66.1 (10.8)	80.9 (10.6)	**<0.01**
	Minute 3	63.1 (10.5)	80.6 (11.9)	**<0.01**	65.3 (11.8)	80.9 (10.1)	**<0.01**
	Mean	60.5 (10.2)	76.4 (9.0)	**<0.01**	62.5 (8.9)	76.4 (9.0)	**<0.01**
Time without compressions (seconds in each CPR session) Mean (SD)		74.0 (12.6)	45.3 (7.1)	**<0.01**	68.4 (18.6)	42.9 (11.3)	**<0.01**

Paired T-student test.

There were no differences in the percentage of optimal compressions in depth between the 15:2 and 30:2 ratios, neither in the child or the infant manikin (Table 2).

**Table 2:** Depth and release velocity of CC.

Parameters		Children manikin			Infant manikin		
		15:2 ratio	30:2 ratio	p	15:2 ratio	30:2 ratio	p
		(n = 26)	(n = 26)		(n = 26)	(n = 26)	
Mean depth (cm) Mean (SD)	Minute 1	4.4 (0.8)	4.4 (0.9)	0.84	3.1 (0.5)	3.1 (0.6)	0.62
	Minute 2	4.4 (0.8)	4.3 (0.8)	0.81	3.0 (0.5)	3.0 (0.6)	0.99
	Minute 3	4.4 (0.8)	4.3 (0.8)	0.45	3.1 (0.5)	3.0 (0.5)	0.07
	Global	4.4 (0.8)	4.3 (0.8)	0.66	3.1 (0.5)	3.0 (0.5)	0.41
Percentage of chest compressions Mean (SD)	Lower depth than optimal	27.7 (37.6)	31.5 (41.3)	0.35	44.9 (39.2)	51.8 (41.3)	**<0.01**
	Optimal depth	48.4 (37.3)	44.1 (38.1)	0.83	52.7 (36.8)	43.7 (38.0)	**<0.01**
	Higher depth than optimal	23.9 (36.4)	24.4 (36.5)	0.94	2.4 (4.9)	4.5 (14.5)	**0.015**
Release velocity (mm/s) Mean (SD)	Minute 1	300.7 (59.4)	296.5 (79.9)	0.71	239.0 (44.3)	243.2 (61.6)	0.011
	Minute 2	284.2 (56.6)	273.2 (64.7)	0.22	226.7 (45.3)	213.8 (44.4)	**<0.01**
	Minute 3	288.9 (58.9)	266.0 (65.1)	**0.017**	223.5 (47.0)	208.8 (41.6)	**<0.01**
	Mean	291.3 (54.4)	278.6 (67.3)	0.13	229.8 (42.7)	221.9 (45.8)	0.23

Paired T-student test.

Deeper of CC was found in the first minute (3.1 ± 0.6) than in the third minute (3 ± 0.5) of resuscitation in infant model with 30:2 ratio (p = 0.018). In the rest of resuscitations, although depth and rate were slightly higher in the first than in the third minute, no significant differences were found with both ratios (15:2 and 30:2) and manikins. No differences were found in rate of CC, nevertheless, the number of CC performed in each minute in the third minute was higher than in the first minute with both compression-to-ventilation ratios and manikins (p < 0.05).

In the infant manikin, 15:2 ratio had higher percentage of CC performed with optimal depth than 30:2. With both manikins the ratio 30:2 achieved a higher number of compressions and less time without compressions than the ratio 15:2. The release velocity was higher with the ratio 15:2 at the end of the resuscitation (Table 2).

### Visual assessment of the quality of resuscitation

**Figure 1:**
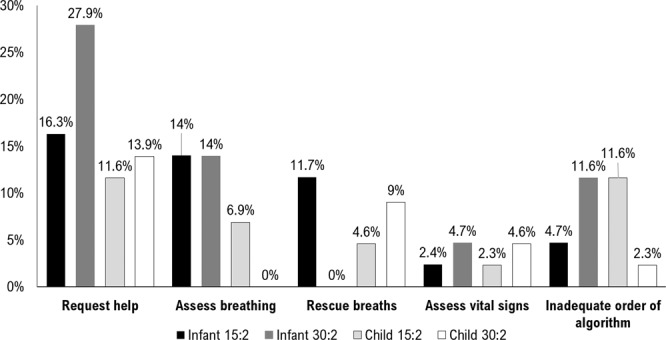
CPR algorithm errors or omissions during resuscitations.

Compliance with European CPR protocol was analyzed: in the child manikin some errors or omissions were found in 69.2% of resuscitations with 30:2 ratio and in 80.8% with 15:2 ratio. In the infant manikin there were CPR algorithm errors in 69.2% of the resuscitations with 30:2 ratio and in 73,1% with 15:2 ratio. Over the half of the volunteers (53,8%) performed at least one CPR session without any errors or omissions, most of them (64,3%), executed without errors more than one session. The most common error in both manikins and both ratios was do not give rescue breaths (23.1%) and not ask for help or do it too late (21.2%). Errors or omissions performed during resuscitation are shown in Fig. 1. No differences were found in errors or omissions between the 15:2 and 30:2 ratio in both manikins.

In the visual assessment of the resuscitation: airway opening in the child manikin was adequate in 65.4% of the resuscitations with 30:2 ratio and in 50% of 15:2 ratio. In the infant manikin it was adequate in 84.6% of the resuscitations with both ratios. Ventilation maneuver in child manikin was adequate in 92.3% of the resuscitations with 30:2 ratio and in 76.9% with 15:2 ratio. In the infant manikin it was adequate in 88.5% with 30:2 ratio and in 80.8% with 15:2 ratio. No differences were found between both ratios for any of the two manikins.

### Preferred compression-to-ventilation ratio

For the child manikin, 65.4% of the rescuers preferred 30:2 ratio. In contrast, for infant manikin, 76.9% of the participants reported that 15:2 ratio was more comfortable because 30:2 produced more weariness of the fingers.

## Discussion

The main objective of our study was to compare CC quality between 30:2 and 15:2 compression-to-ventilation ratio in pediatric and infant manikins. In our study, there were no differences in the rate or depth of CC between the ratio 15:2 and 30:2. A high percentage of compressions were not performed with optimal rate and depth. Furthermore, depth and rate of CC were not modified throughout the CPR session. The results could not allow to conclude that a compression-ventilation ratio is better than the other. Our study highlights the relevance of periodic recertifications.

No differences were found in depth or rate of CC in both manikins between 15:2 and 30:2 compression-to-ventilation ratio. In our knowledge, there are no studies in pediatric CPR comparing CC quality in 15:2 or 30:2 compressions-to-ventilations ratios developed by volunteers without PALS accreditation. There is only one study^11^ that compared, in manikin-simulated pediatric resuscitation, three compressions-to-ventilations ratios (5:1, 10:2 and 15:2). In this study there were no differences in depth and rate between the three ratios.

Although depth and rate of chest compressions is the same with both compression-to-ventilation ratios, it is not clear whether survival increases with increasing number of ventilations or prioritizing chest compressions. In a study of adults with out-of-hospital cardiac arrest, continuous CC without ventilations did not result in significantly higher rates of survival or favorable neurologic function than 30 compressions to two ventilations^16^. In other similar study the percentage of one-month survival patients with good neurological outcome was lower with continuous CC, however, in the multivariate analysis the continuous CC group showed better neurological outcome than the CC plus ventilation group^17^. In a pediatric asphyxial arrest animal model^18^ CC plus ventilation produced better oxygenation, ventilation, and cerebral oxygenation than compression-only CPR. In observational studies of children resuscitation who had out-of-hospital CA^19,20^ CPR with CC plus ventilation produced better outcomes than compression-only CPR. The fact that the main cause of pediatric CA is respiratory failure could be the reason for better outcomes of CPR with CC plus ventilations than compression-only CPR.

We found slightly higher chest compression release velocity with 15:2 ratio but these differences are not relevant. One study showed the association of chest compression release velocity with higher survival and favorable neurologic outcome after out-of-hospital cardiac arrest in adults patients^21^. In this study the adjusted odds of survival increased from slow (<300 mm/s) to fast release velocity (≥400 mm/s) and from moderate (300–399.9 mm/s) to fast (≥400 mm/s) release velocity. It should be noted that in our study the release velocity in all groups oscillated between 220 and 300 mm/s, corresponding in all cases with the slow velocity. However, the influence of CC release velocity and survival is still controversial because other studies have found no relationship between them^22^. More studies are needed to analyze the role of this parameter in the quality of resuscitation.

There is good evidence supporting the use of CPR feedback/prompt devices during CPR training to evaluate and improve CPR quality^23^. In our study, we use one device to analyze CC quality. We also used video recordings to asses pediatric resuscitation management and to detect errors or omissions during resuscitation because previous studies showed that it is a useful tool for this purpose^24^.

Some studies have investigated optimal depth of CC but there is a lack of evidence in infants and children CC^25–27^. Although deeper CC have shown higher arterial blood pressure^28^, excessive CC depth may cause serious mechanical complications^25^. In both manikins but, especially, in the infant model, high percentage of CC were shallower than recommended. This fact may be due to the difficulty of performing CC in an infant manikin encircling the chest and compressing only with the thumbs. Smereka and Ladny designed a new technique for chest compressions in infants using two thumbs directed at the angle of 90 degrees to the chest while closing the fingers of both hands in a fist. They described this new technique and compared it in two studies performed on manikins with those maneuvers used routinely^29,30^ showing that a higher simulated blood pressure was reached with this new technique^29,30^. As in our study, other studies that have analyzed the quality of CC in pediatric manikins, found a depth of CC lower than recommended^7^. Therefore, shallow CC could also be related to low fidelity of the manikin used.

Vaillancourt *et al*.^13^ developed an adult simulated CPR manikin cross-over study with elderly volunteers that compare 15:2 to 30:2. This study showed that the 15:2 ratio resulted in proportionally more adequate compressions (defined as depth of 4–5 cm followed by full decompression during each minute). They used a metronome to encourage the administration of chest compressions at a rate of 100 per minute, so rate of CC should not be evaluated.

In our study, in both compression-to-ventilation ratios, an important percentage of CC were performed with a higher or lower rate than optimal (>120 or <100 cpm). It should be noted that, although half of CC were performed with optimal rate, the number of CC performed in each minute is not high, which can be explained by the time used for ventilation. With the ratio 30:2 the number of CC performed was higher than the ratio 15:2. In a study by Haque *et al*.^15^ more compression cycles were achieved with 30:2 ratio without effect in compression depth and rate, similar to our results. However, they reported higher subjective fatigue in the 30:2 ratio.

Depth and rate of chest compressions did not change significantly during the three minutes of CPR with any of the compressions-to-ventilation ratios in our study. This might have been because each CPR session lasted only 3 minutes. Nevertheless, other published studies with simulated CPR sessions and shorter periods found a decrease in depth over time^26^. Vaillancourt *et al*.^13^ measured objective fatigue by changes in heart rate, mean arterial pressure and venous lactate, and perceived level of exhaustion using the validated Borg Rating of Perceived Exertion scale. They have found that the 30:2 ratio resulted in similar objective measures of fatigue, but higher perceived fatigue than the 15:2 ratio. In our study, 60% of the rescuers felt that 30:2 ratio was more comfortable than 15:2 in the child model. This could be due to the smaller number of position changes for ventilation that needs to be done in 30:2 ratio. In contrast, 82% of the volunteers reported that the 15:2 ratio was more comfortable for infant CPR, attributing it to the fact that the 30:2 ratio caused greater weariness of the fingers.

We have observed that, despite having done a pediatric CPR course in the last year, a high percentage of rescuers made some errors or omissions in CPR algorithm, the most frequent giving five initial recue breaths and forgetting to ask for help. Rescuers who performed without error or omissions one CPR session, usually did it without errors in another session. This may be because some volunteers internalize knowledge better than others. There is no agreement in which the best time interval for doing CPR recertification courses is and the best method to keep the skills acquired. Traditionally it has been recommended that recertification should be done every 1 or 2 years at most^31^. Our results highlight the importance of frequent refreshment of CPR training, possibly between 6 months and 1 year.

The study is a pediatric simulation model with manikins that compare to compression-to-ventilation ratios. Experimental or clinical studies should be carried out, in which other factors that may affect survival, such as ventilation, oxygenation, blood pressure, etc., will be analyzed in addition to the quality of CC.

The scope of the study is limited to single rescuer CPR. It could be different in situations in which two rescuers are providing CPR. The pauses of transitions between compressions and ventilations by a single rescuer should be longer than when CPR is performed by more than one rescuer. The study also has the inherent limitations of those performed with manikins. Experimental and clinical studies are needed to analyze the effect of the two compression-to-ventilation ratios and the interaction between quality of chest compressions and ventilation and oxygenation achieved during resuscitation. Other potential limitation was that the researchers could not be blinded to resuscitation method and the small sample size. The assessment of the resuscitations maneuvers as opening of the airway and ventilation was carried out subjectively, although evaluation criteria were defined previously. The qualitive assessment was performed by the same researcher to avoid interobserver variability. Finally, in our study participant expressed their preference between 15:2 or 30:2 ratio from and subjective point of view, but no other objective fatigue scale was used as in Vaillantcourt^13^ study.

## Conclusions

In this model of pediatric CPR simulation, a high percentage of chest compressions were not performed with optimal frequency and depth. No differences were found in depth or rate of CC between 15:2 and 30:2 compression-to-ventilation ratio with both manikins. Depth and rate of CC were not modified during de CPR session. The participants preferred 30:2 ratio in the child manikin and 15:2 ratio in the infant manikin. Approximately three quarters of the participants performed errors or omissions during resuscitation.
